# The Mediating Role of Critical Thinking Abilities in the Relationship Between English as a Foreign Language Learners’ Writing Performance and Their Language Learning Strategies

**DOI:** 10.3389/fpsyg.2022.746445

**Published:** 2022-02-21

**Authors:** Maryam Esmaeil Nejad, Siros Izadpanah, Ehsan Namaziandost, Behzad Rahbar

**Affiliations:** ^1^Department of English Language Education, Islamic Azad University, Zanjan Branch, Zanjan, Iran; ^2^Assistant Professor of Applied Linguistics, Islamic Azad University, Zanjan Branch, Zanjan, Iran; ^3^Department of English, Islamic Azad University, Shahrekord Branch, Shahrekord, Iran; ^4^Assistant Professor of Linguistics, Faculty of Persian Literature and Foreign Languages, Allameh Tabataba’i University, Tehran, Iran

**Keywords:** EFL learners, critical thinking, language learning strategies, mediating role, writing performance

## Abstract

Recent developments in the field of education have led to a renewed interest in the mediating role of critical thinking abilities (CTA) in the relationship between language learning strategies and the intermediate English as a Foreign Language (EFL) learners’ writing performance. Oxford Placement Test (OPT) was run to homogenize the participants, and 100 intermediate learners out of 235 were selected. Then, two valid questionnaires of Ricketts’ Critical Thinking Disposition and Oxford’s Strategy Inventory for Language Learning were administered. Having administered the questionnaires, the researchers asked the participants to sit for a writing test. The data collected from the questionnaires and as well as the scores of their writing performances were analyzed through SPSS (25.00). The results showed a significant relationship between (a) learning strategies and learners’ writing performances, (b) the sub-sets of learning strategies and learners’ writing performances, and (c) CTA and learners’ learning strategies. However, CTA did not play a mediating role in the relationship between intermediate EFL learners’ learning strategies and writing performance. Based on the results of the study, one might also conclude that strategies seemed to play a more important role in the performance of learners especially their writing performances. Therefore, this study had useful contributions for students, teachers, and curriculum designers. Findings of this research could assist teachers to be aware of learners’ strategies in learning writing and help their students to be responsive to using learning strategies in their learning process and create a satisfactory learning context for using learning strategies. Therefore, learners were able to become independent and feel responsibility for their own learning. Secondly, curriculum developers could take advantage of the findings to include learning strategies training into the curriculum. As a result, students were able to use strategies in their learning process more easily and finally, the results might pave the way for improving the research findings.

## Introduction

This study examined the relationships between writing performance and language learning strategies (cognitive, metacognitive, social, affective, memory-related, and compensation). It also intended to determine the relationship between intermediate English as a Foreign Language (EFL) learners’ critical thinking abilities and their writing performances. There are some factors which could affect language learning strategies by learners. According to some researchers’ different variables such as age, gender, language proficiency, motivation, anxiety, aptitude, and cultural background affect using language learning strategies by students (e.g., Berridge, 2018; Esteves et al., 2021). However, a quick review of the global literature shows that little attention is given to the role of critical thinking in learners’ choice of learning strategies as well as their writing performances.

Recent developments in the field of critical thinking abilities have also led to a renewed interest in EFL learners’ writing performance (Renatovna and Renatovna, 2021). It is becoming increasingly difficult to ignore the role of critical thinking abilities (Warsah et al., 2021). Recently, the focus of the teacher-oriented viewpoint has changed to a learner-centered perspective (Reshadi and Aidinlou, 2012; Al Sharadgah, 2014; Yaralı and Aytar, 2021). Learners are now in charge of their learning more than ever. In other words, more learners take more responsibility to make the best use of language learning strategies (LLSs) and be conscious of their own individual needs (Teng, 2020; Parra et al., 2021). New learning strategies are introduced to the learners to develop their personable attainments in the language learning process (Sutiani et al., 2021).

Learning strategies are made up of mental processes – thoughts or behaviors – that help learners understand, learn, or sustain new information (Panahandeh and Esfandiar, 2014; Jiang et al., 2021). The concept of learning strategies plays a crucial role in the study of second or foreign language learning. Even though many studies have been done to implement learning strategies, the idea of learning strategies is still obscure. Dörnyei and Skehan (2003), Rajaee Pitenoee et al. (2017), and Chang et al. (2021) assert that the opinion behind learning strategies has not been critically examined because the concepts and definitions have been inconsistent so far. Critical thinking abilities play a significant role in language learning and teaching. Choosing critical thinking abilities, among other skills and strategies, is to help students do writing performance difficulties and teachers who try to help do their students’ writing performances.

In addition, few studies have been carried on to compare the learning strategies of cognitive, metacognitive, and social/affective, memory-related strategies with writing performances. Besides, no studies, to the best knowledge of the researchers, had ever embarked on investigating the mediating role of critical thinking abilities in the relationship between intermediate EFL learners’ writing performance and their learning strategies. A quick review of the global literature shows that little attention is given to the role of critical thinking abilities in learners’ choice of learning strategies and their writing performances.

In a nutshell, this study aimed to evaluate the mediating role of critical thinking abilities in the relationship between EFL learners’ writing performance and their language learning strategies.

## Review of Literature

### Learning Strategies and Writing Performance

Learning strategies are defined as “proceedings or stages used by a learner to comfort the attainment, storage, detection or use of information” (Rigney, 1978 cited in Aslan, 2009, p. 45). O’Malley and Chamot (1990) defined learning strategies as the particular thoughts or compartments that everybody uses to understand, learn, or maintain new information. On the other hand, Chamot (2004), Bagheri (2015), and Zarrinabadi et al. (2021) claim that learning strategies are the purposive thoughts and behavior that students take to earn a learning goal. More importantly, successful learners have their unique techniques to learn. Rubin (1975) and Stern (1975) have been the first scholars who analyze the idea of successful language learning. This idea makes us more curious to discover more about the nature of the learning and learning process. Consequently, the majority of the research performed until now has been focusing on the detection, explanation, and categorization of learning strategies (Pradhan and Das, 2021; Tran and Tran, 2021).

The relationship between language learning strategies (LLS) and writing performance (WP) has been the subject of much research over the last 20 years (Goudarzi et al., 2015; Wale and Bogale, 2021). According to Green and Oxford (1995), the picture is not crystal clear because a lot of research has focused on overall strategy use only and not considered individual strategy use or variations. In a study done by Saricoban and Saricaoglu (2008) in Turkey, it was found that compensation strategies had a positive correlation with academic achievement (p. 172) while affective strategies were negatively correlated. Students who used affective strategies were less successful than others. Griffiths (2004), in a study at a private language school in Auckland, found that “there was a significant relationship between strategy use and language proficiency” (p. 82). The study showed that the “Advanced students reported a higher average frequency of use of each strategy than did elementary students” (p. 78). These studies imply that we can raise levels of proficiency by teaching these strategies. These studies may not have shown a clear causality in any direction between language proficiency and strategy use; however, it can be logically concluded that there are significant relationships between the two.

According to Oxford (1990), learning strategies are categorized into direct and indirect strategies. Also, each category is divided into subcategories which are placed under the labels. Learners directly use direct strategies in the learning process to produce the target language. These strategies include memory strategies which are responsible for retrieving and storing information, cognitive strategies which learners use to process new information; and learners use compensation strategies to compensate for lack of enough knowledge in the target language (Goudarzi et al., 2015; García-Sánchez and García-Martín, 2021; Zarrinabadi et al., 2021). As Oxford (1990) states, these strategies assist students to be more independent, identify their learning strengths and weaknesses, and be self-reliant in their language learning process. Therefore, learning strategies help learners to become competent in using a language. Based on Oxford and Burry-Stock (1995), strategies are techniques or behaviors used unconsciously by learners to improve their understanding and use the target language. O’Malley and Chamot (1990) proposed a very comprehensive classification of learning strategies. Their tri-faceted classification is as follows:

1. Metacognitive strategies: It includes supervisory processes in planning for learning, supervising one’s understanding and production, and assessing to what extent individuals have achieved a learning goal.
2. Cognitive strategies: Mentally speaking, manipulating the materials to be learned through interaction by visualizing mental pictures or connecting the material with the previously known items. Physically speaking, categorizing the things to be learned meaningfully or summarizing the essential items to be known.
3. Social-affective strategies: Learner’s interact with others to look for help in learning, such as posing questions for cooperation or using some affective to control learning.

### Critical Thinking Abilities and Writing Performance

In modern society, even in everyday life, people frequently need to deal with complicated public and political issues, make decisions, and solve problems (Bagheri, 2015; Zarrinabadi et al., 2021). To do this efficiently and effectively, citizens must evaluate critically what they see, hear, and read. Although a massive amount of printed material is available in all areas in the age of “information explosion,” it is still easy to feel overwhelmed. But the information piled up on people’s desks and in their minds is of no use due to the enormous amount of it. Thus, they need to read selectively and sort out the bits and pieces that are interesting and useful for them. To do so, strong critical reading and critical thinking skills are indispensable (Morgan and Shermis, 1994).

Writing is a complex process that needs much effort to be completed. Numerous researchers believe that writing is a skill that requires learning and practicing (Fathi et al., 2019; Neimaoui, 2019; Wale and Bogale, 2021). Also, Langan (1987), Reid (1993), and El-Freihat and Al-Shbeil (2021), note that writing is a craft skill that can be taught and learned. For effective writing in EFL classrooms, ELT practitioners (Badger and White, 2000; Paltridge, 2004; Rahayu, 2021) suggest three following approaches: product, process, and genre. According to Zamel (1983), Liu and Hansen (2002), and Indah (2017), the process approach focuses on the composing process, whereby writers express their notions as they attempt to transfer the meaning. According to Gabrielatos (2002) and Hall (2017), a product approach is a traditional approach, in which students are motivated to copy a model text while the genre approach is the newcomer and an outcome of the communicative language teaching approach. The readers are at the center of this approach since its readership must successfully accept it.

The word “writing” means the text in written form in the process of thinking, constructing, and coding language into such text (Tabibian and Heidari-Shahreza, 2016; Irzawati et al., 2021; Namaziandost et al., 2021). Since writing is one of the skills in first and second language learning, all skills have a relationship. As an instance, Harmer (1991), Rahimi and Karbalaei (2016), Taghinezhad et al. (2018), and Yan (2018) believe that one skill cannot be carried out without the other, and it is impossible to communicate without listening, and people seldom write without reading.

The relation between writing and thinking is that writing is thinking if one cannot think clearly, one cannot write clearly. Writing develops thinking skills. It improves the thinking process and contributes to the development of thinking skills because an individual has to clearly state ideas and lay out arguments in such a way as to cultivate higher order of thinking. Regarding the relationship between both, the Sapir-Whorf hypothesis (1956 in Errihani, 2012; Jiang et al., 2021; Wale and Bogale, 2021) is suggestive in the context of English as a Foreign Language as it contends that cognitive activity is determined by language. The cognitive activity can be reflected in written text and later be understood well by the audience determined by the strength of the language (Díaz Larenas et al., 2017; Rahayu, 2021). Consequently, the primary concern of second language (L2) writers is primarily on linguistics, as noted by Errihani (2012). Therefore, critical thinking ability reflects their linguistic skill represented by their writing, which reflects the background knowledge.

### The Relationship Between Critical Thinking Abilities and Learning Strategies

Literature on the relationship between critical thinking and language learning strategies is not much. However, a number of studies have been conducted so far. In a survey conducted by Nikoopour et al. (2011), they surveyed the relationship between CTA and the use of LLS by Iranian language learners. Their findings reveal a significant correlation between direct and indirect LLS such as cognitive, meta-cognitive, and social with critical thinking. At the same time, no relationship was discovered between CTA and memory, compensation, and affective strategies. In another study by Ku (2009), they aimed to examine the role of meta-cognitive strategies in critical thinking. Based on the findings, “good critical thinkers” are more active in meta-cognitive activities.

Although critical thinking ability is not directly measurable and is not easy to teach, there is always a chance to enhance these strategies through deliberate teaching (Willingham, 2007; Nikoopour et al., 2011; Mosley et al., 2016). Learning strategies can develop and improve it (Loving and Wilson, 2000; Seymour et al., 2003). The teacher is responsible for its development (Choy and Cheah, 2009). Willingham (2007) stated that one of the fundamental purposes of education is to enable students to think critically, but this goal is incompetently met. As the 21st century is the age of information technology, critical thinking abilities are a crucial requirement to select and evaluate the reliability of the information (Grabau, 2007). Asian students lack the required skill as it is not commonly emphasized in schools (Egege and Kutieleh, 2004; Djiwandono, 2013). Learning activities have been used to develop the critical thinking skills of the learners for years. Literature suggests cooperative learning is very fruitful for developing students’ social skills, language acquisition, and academic achievement and fostering critical thinking skills (Ghaith, 2003; Sadeghi, 2012). Students who learn through strategies have a chance to develop their thinking. Students’ face-to-face interaction promotes critical thinking abilities (Fahim and Eslamdoost, 2014). Group discussions effectively stimulate and develop ideas, which is the first requirement of critical thinking abilities (Devi et al., 2015). The student’s critical thinking abilities can be enhanced through cooperative learning. In collaborative learning, students have a chance to group discussion, evaluate and synthesize the information, and consider the solution as students are responsible for their learning. Cooperative learning promotes interaction among students, which helps develop critical thinking abilities (Devi et al., 2015; Mahmoodi and Dehghannezhad, 2015).

This study examined the relationships between writing performance and language learning strategies (cognitive, metacognitive, social, affective, memory-related, and compensation). This study also intended to determine the relationship between intermediate EFL learners’ critical thinking abilities and their writing performances. Based on the analysis, one might also conclude that strategies seemed to play a more important role in learners’ performance, especially their writing performances. Therefore, this study had valuable contributions for students, teachers, and curriculum designers.

All in all, reviewing the literature so far indicates that the impact of CTA on language skills and sub-skills has not received as much attention as warranted. Moreover, rare studies, if any, have been done in this regard Iranian context. Thus, to cover these gaps, the researchers aim to explore if CTA has any role in the relationship between language learning strategies and the intermediate EFL learners’ writing performance. To this purpose, the following research questions were proposed:

1. Is there any significant relationship between intermediate EFL learners’ learning strategies and their writing performances?
2. Is there any significant relationship between intermediate EFL learners’ cognitive strategy (CS), metacognitive strategy (MS), social strategy (SS), affective strategy (AS), compensation strategy (CS), memory-related strategy (MS), and their writing performances?
3. Is there any significant relationship between intermediate EFL learners’ critical thinking abilities and writing performances?
4. Is there any significant relationship between intermediate EFL learners’ learning strategies and their critical thinking?
5. Does critical thinking ability play a mediating role in the relationship between intermediate EFL learners’ learning strategies and writing performances?

## Materials and Methods

### Participants

The participants of this study were 235 male and female Iranian EFL learners at different language institutes in Zanjan, Iran who were selected based on the convenience sampling method. Oxford Placement Test (OPT) was run to make the participants homogeneous, and 100 learners were selected as the final participants. According to the OPT, 37 people had advanced level scores and 98 people had elementary level scores who were excluded from the study. That is, low- and high-level average scores based on the OPT were summarized and included in the study [Mean (SD) = 37.5 ± 9.15]. The selected participants were all EFL intermediate learners at language institutes ranging from 18 to 35 years of age. More details about the participants can be seen in Table 1.

**TABLE 1:** Demographic characteristics of the participants.

	Number	Age	Gender	Level of proficiency	First language
Participants	100	18–35	Males (50); Female (50)	Intermediate	Persian

### Instruments

In line with the purposes of this research, three instruments were used:

*(1) OPT:* To meet the purposes mentioned above, at first, a language skill test version 2, including 60 items matching cloze passages and multiple-choice questions were managed to sure the concord of the learners. The test items most focused on grammar and vocabulary. The participants were given 30 min to answer. Those learners whose scores fell between 30 and 39 were considered intermediate ones.
*(2) Critical Thinking Dispositions Questionnaire* (Ricketts, 2003)*:* Used to measure the intermediate EFL learners’ critical thinking disposition. The questionnaire contained 33 statements on the Likert 5-point scale. The minimum mean and maximum scores that could be achieved were 33, 99, and 145. Three sub-components of the questionnaire are creativity with 11 sentences, sophistication with nine statements, and dedication with 13 statements. The Cronbach’s alpha coefficients for the invention, sophistication, and commitment subcomponents are 0.64, 0.53, and 0.82, respectively. The reliability coefficient of the instrument was stated to be approximately 0.76 by PakMehr et al. (2010).
*(3) Strategy Inventory for Language Learning Questionnaire* (Oxford, 1990)*:* The following inventory included in this analysis was the Strategy Inventory for Language Learning (SILL) questionnaire used to classify LLS students. The SILL questionnaire was developed by Oxford (1990) and was used without alteration in this research. It comprised 50 items that included six types of LLSs: recall strategies, cognitive strategies, compensation strategies, metacognitive strategies, affective strategies, and social strategies. The questionnaire was a 5-point Likert scale that ranged from 1 (*Never or almost never true of me*), 2 (*Usually not true of me*), 3 (*somewhat true of me*), 2 (*usually true of me*), and 5 (*always or almost always true of me*).

### Writing Performance

In order to measure the writing performance of the participants, they were asked to sit for a writing exam in the class. An argumentative topic titled “Using *a computer every day can have more negative than positive effects on your children. Do you agree or disagree?”* was introduced to the intermediate learners to compose a well-formed essay.

There were many different types of rubrics in the literature for assessing writings. One of the appropriate scales for rating the writing of learners was Cooper’s (1997) scale. This rubric includes different criteria for assessing learning writing performance. Cooper’s (1997) checklist is shown below:

Rating scales covered “Task Achievement,” “Coherence and Cohesion,” “Lexical Resource,” and “Grammatical Range and Accuracy.”

**FIGURE 1:**
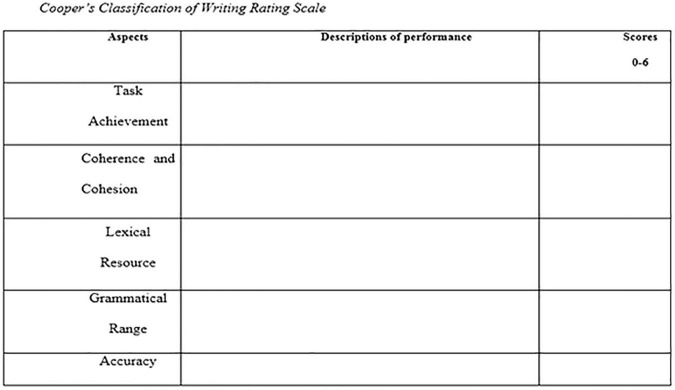
Cooper’s classification of writing rating scale. 6, outstanding; 5, very good; 4, good; 3, adequate; 2, less than adequate; 1, poor; 0, no substantive response.

In the holistic grading method, as illustrated in Figure 1, the reader assigns a single score from 0 to 6 (0, 1, 2, 3, 4, 5, or 6) to an essay based on overall writing quality.

Each essay was scored based on the four scales of “Task Achievement,” “Coherence and Cohesion,” “Lexical Resource,” and “Grammatical Range,” and “Accuracy.” In the end, the average of the five scales showed the last score given to any essay by each rater. Subsequently, the average score given by the two raters represented the writer’s final score.

### Procedure

In order to obtain fair answers to the study questions alluded to above, the following steps have been taken. First, the OPT was spread among EFL students from different institutes in Zanjan to assess the participants’ homogeneity and choose advanced language learners. One hundred participants receiving scores from 30 to 39 were selected as the final sample. Second, the Critical Thinking Dispositions Questionnaire (CTDQ) (Ricketts, 2003) was distributed among the intermediate EFL learners. The CTDQ questionnaire included 33 Likert items. Moreover, the Approach Inventory questionnaire (Oxford, 1990) for language learning was administered to the selected intermediate learners. It was a 50-item questionnaire with a 5-point Likert scale ranging from 1 (never or almost never applies to me) to 5 (always or almost always applies to me).

Afterward, the participants were asked to sit for an essay writing test. An argumentative topic entitled “Using *a computer every day can have more negative than positive effects on your children. Do you agree or disagree?”* was introduced to the intermediate earners to compose a well-formed essay on. Two raters scored all the essays based on Cooper’s (1997) rubric scale. The average score given by the two raters accounted for the learners’ final writing score. In the end, the scores of their writing performances and the data gathered from the SILL and Critical Thinking Disposition questionnaires were put into SPSS version 25 to be calculated.

The study was an ex-post-facto design since there were no treatments at all. Having collected the results, the researchers recorded the scores in computer files for statistical analysis using the Statistical Package for the Social Sciences (SPSS) version 25.0. After homogenizing the students as intermediate, to measure the relationship between the variables (SILL and SILL components and writing performance), since the normality was met, seven Pearson correlation tests were conducted. In addition, another Pearson correlation test was run to find if there was a significant relationship between critical thinking abilities and writing performance. Furthermore, the Pearson correlation test was conducted to measure the relationship between learning strategies and intermediate EFL learners’ critical thinking abilities as a whole. Finally, to figure out whether critical thinking abilities would play a mediating role in the relationship between learning strategies and writing performance, the Sobel test was used.

## Results

This study aimed at investigating the mediating role of critical thinking abilities in the relationship between learning strategies, including cognitive, meta-cognitive, memory-related, compensation, social, and affective strategies, and intermediate EFL learners’ writing performance. First of all, it was necessary to check the normality distribution. Thus, a One-Sample Kolmogorov–Smirnov Test was run.

Based on the statistics in Table 2, all the *p*-values are higher than 0.05 (*p* > 0.05), it, thus, can be concluded that all the variables benefit from a normal distribution. Accordingly, the researchers are allowed to utilize parametric analysis of the data. The research questions of this study are answered in this part. The related descriptive analysis of all variables will be discussed before defining the inferential analysis:

**TABLE 2:** Normality tests: One-sample Kolmogorov–Smirnov test.

Variables	Sig.	Decision	Test result
Total strategies	0.163	The null hypothesis is accepted	Distribution is normal
CS	0.097	The null hypothesis is accepted	Distribution is normal
MS	0.189	The null hypothesis is accepted	Distribution is normal
SS	0.20	The null hypothesis is accepted	Distribution is normal
AS	0.20	The null hypothesis is accepted	Distribution is normal
CS	0.095	The null hypothesis is accepted	Distribution is normal
MS	0.183	The null hypothesis is accepted	Distribution is normal
CT	0.20	The null hypothesis is accepted	Distribution is normal
Writing performances	0.112	The null hypothesis is accepted	Distribution is normal

As Table 3 shows, regarding the number of participants (*N* = 100), the mean and the SD of writing performance are 4.22 and 3.27, respectively. The means for critical thinking abilities and learning strategies are 112.56 and 195.20, respectively.

**TABLE 3:** Descriptive statistics of the research variables.

	N	Minimum	Maximum	Mean	Std. deviation
Total strategies	100	60.00	235.00	195.20	3.21
CS	100	21.00	65.00	56.25	2.36
MS	100	13.00	42.00	32.28	1.62
SS	100	8.00	27.00	23.65	2.39
AS	100	9.00	28.00	25.45	2.85
CS	100	8.00	25.00	22.91	5.17
MS	100	11.00	42.00	34.62	3.09
CT	100	45.00	136.00	112.56	2.99
Writing performances	100	2.00	6.00	4.22	3.27

First of all, regarding the relationship between intermediate EFL learners’ learning strategies and their writing performances, the results of Pearson correlation displayed in Table 4 [*r*(98) = 0.865, *p* < 0.05 representing a large effect size] it can be concluded that there was a significant relationship between learning strategies and writing performance.

**TABLE 4:** Pearson correlation: Learning strategies with writing performances.

		Learning strategies
Writing performances	Pearson correlation	0.865
	Sig. (two-tailed)	0.000
	N	100

Moreover, considering the relationship between intermediate EFL learners’ CS, MS, SS, AS, CS, MS, and their writing performances, a Pearson correlation was run, which shows that there was a significant relationship between EFL learners’ CS, MS, SS, AS, CS, MS, and their writing performance (*p* < 0.05) (Table 5).

**TABLE 5:** Pearson correlation: CS, MS, SS, AS, CS, and MS with writing performances.

		CS
Writing performances	Pearson correlation	0.668
	Sig. (two-tailed)	0.000
	N	100

		**MS**

Writing performances	Pearson correlation	0.872
	Sig. (two-tailed)	0.000
	N	100

		**SS**

Writing performances	Pearson correlation	0.775
	Sig. (two-tailed)	0.000
	N	100

		**AS**

Writing performances	Pearson correlation	0.790
	Sig. (two-tailed)	0.000
	N	100

		**CS**

Writing performances	Pearson correlation	0.767
	Sig. (two-tailed)	0.000
	N	100

		**MS**

Writing performances	Pearson correlation	0.765
	Sig. (two-tailed)	0.000
	N	100

In addition, to check the relationship between intermediate EFL learners’ critical thinking abilities and their writing performances, the Pearson correlation analysis in Table 6 [*r*(98) = 0.843, *p* < 0.05] indicates that the relationship between critical thinking abilities and writing performance was significant.

**TABLE 6:** Pearson correlation: CTA with writing performances.

		CTA
Writing performances	Pearson correlation	0.843
	Sig. (two-tailed)	0.000
	N	100

Furthermore, regarding the relationship between intermediate EFL learners’ learning strategies and their critical thinking abilities, Pearson correlation results show a significant relationship (*p* < 0.05) (Table 7).

**TABLE 7:** Pearson correlation: CTA with learning strategies.

		Learning strategies
CT	Pearson correlation	0.946
	Sig. (two-tailed)	0.000
	N	100

Lastly, considering the relationship between intermediate EFL learners’ learning strategies and their writing performances, the following conceptual model was used to illustrate the direct impact of learning strategies on writing performance.


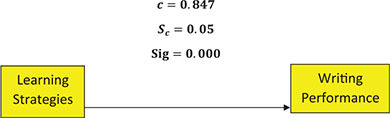


As it is shown, the direct impact of learning strategies on writing performance is 0.847, with the SD of 0.05. Regarding the fact that *p* < 0.05, it is believed that direct impact is significant. The conceptual model below shows the mediating role of critical thinking abilities in the relationship between learning strategies and writing performance:


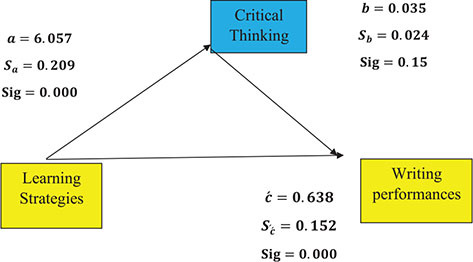


As depicted above, the impact of the mediating role of CTA on writing performance was 0.035, with the SD of 0.024. Since *p* > 0.05, it is concluded that the impact of CTA on writing performance is not significant. As also illustrated above, the impact of learning strategies on CTA is 6.057, with the SD of 0.209. Because *p* < 0.05, it is concluded that the impact of learning strategies on CT is significant. Besides, the impact of learning strategies on writing performance, with the mediating role of CTA, is 0.638, SD = 0.152. Regarding the *p*-value, which is less than 0.05 (*p* < 0.05), it is concluded that the impact of learning strategies on writing performance, with the mediating role of CTA, is not significant because the *Z* value of the Sobel test is 1.46 and *p* > 0.14. Thus, CTA does not play a mediating role in the relationship between learning strategies and writing performance.

## Discussion

As stated earlier, this study made an effort to find answers to all research questions concerning the relationship between the three variables CTA, learning strategies, and writing efficiency. Reasonable methods have been used to obtain the answers needed for each question. The study found that high CTA students outperformed low CTA students. Cognitive and metacognitive techniques are not independent; they operate together as learners undertake the process of writing.

Both learning strategies and CTA played an essential role in students’ writing performance. CTA is highly related to writing performance, and it was proved those with high CTA did better in their writing performance, especially when the subject was a bit controversial. Furthermore, it was shown that utilizing learning strategies would improve learners’ writing performance. In other words, the more use of strategies, the better scores in writing performance.

As seen before, the relationship between intermediate EFL learners’ learning strategies and their writing performance was significant. In addition, the relationships between intermediate EFL learners’ learning strategies subsets, including cognitive, metacognitive, affective, social, memory-related, and compensation strategies, and their writing strategies were all significant, meaning all the six subsets were significantly correlated with writing performances. The result of this study is in line with the studies done by Chamot (2004), Berridge (2018), Al-Jarrah et al. (2019), Teng (2020), Chang et al. (2021), and Parra et al. (2021) in that metacognitive strategies together with its subsets of planning, organizing, and evaluating strategies are related to EFL learners’ writing performances. Besides, this study also confirmed the findings of Pradhan and Das (2021) and Tran and Tran (2021) that there was a positive correlation between English academic achievement and metacognition. In harmony with Teng (2020) and Jiang et al. (2021), metacognitive strategies could yield the highest mean scores of EFL learners’ writing performances.

In congruence with the finding of Yan (2018), García-Sánchez and García-Martín (2021), and Parra et al. (2021), this study showed a positive correlation between cognitive and metacognitive strategies of learners with their writing performances. Compatible with the findings of Díaz Larenas et al. (2017) and Rahayu (2021), this study also showed that metacognitive and cognitive strategies would benefit EFL learners’ writing performance.

As Díaz Rodríguez (2014) asserts, this study also illustrated that cognitive and metacognitive strategies are not independent from one another; they work together while the learner is performing the task of writing. Following Rajaee Pitenoee et al.’s (2017) outcome, cognitive and metacognitive strategies would affect Iranian intermediate EFL learners’ writing performance. Meanwhile, in line with Azizi et al. (2017), this study also confirmed that metacognitive strategies would contribute to higher proficiency in writing. In addition, as Tabrizi and Rajaee (2016) and Tran and Tran (2021) put forward, this study also concluded that cognitive and metacognitive writing strategies would help learners improve their writing. Besides, in agreement with Panahandeh and Esfandiar (2014), this study showed that metacognitive strategies were positively correlated with writing performance.

However, as opposed to Rahimi and Karbalaei (2016), who did not find any relationship between the use of metacognitive strategies and writing performance of EFL Iranian learners, this study concluded that metacognitive strategies were highly correlated with writing performance. Compatible with the findings of Goudarzi et al. (2015) and Wale and Bogale (2021), the results of this study depicted that metacognitive awareness strategies highly affect achievement scores, and there was a significant correlation between metacognitive awareness strategies and their task performance.

Concerning the relationship between intermediate EFL learners’ CTA and their writing performance’, it was revealed that intermediate EFL learners’ critical thinking abilities were significantly correlated with writing version. As Neimaoui (2019), Renatovna and Renatovna (2021), and Saenab et al. (2021) claim, this study justified that critical thinking ability plays a significant role in EFL learners’ writing performances. Furthermore, consistent with the finding of Hall (2017) and Warsah et al. (2021), this study concluded that critical thinking abilities could lead to an improvement in EFL learners’ writing performances. Moreover, in agreement with Indah (2017) and Yaralı and Aytar (2021), this study also stated that EFL learners’ writing performance was influenced by critical thinking. Furthermore, in line with Al Sharadgah (2014), this study also depicted that those benefiting from a high level of critical thinking abilities would show a more remarkable improvement in their writing. In line with Taghinezhad et al. (2018), this study proved that critical thinking abilities would improve students’ writing performance. This study also corroborated Golpour’s (2014) finding that critical thinking abilities would play a crucial part in learners’ writing performance. In other words, high critical thinkers were better in writing compared to low critical thinkers.

Considering the relationship between intermediate EFL learners’ learning strategies and their critical thinking, it was shown that these two variables were significantly correlated. This study is in line with Bagheri (2015) and Zarrinabadi et al. (2021), who reported a significant relationship between CTA and language learning strategies. Besides, this study also confirmed Nikoopour et al. (2011). They surveyed the relationship between CTA and the use of language learning strategies by Iranian language learners. Their findings revealed a significant correlation between cognitive, meta-cognitive, and social strategies with critical thinking. However, as opposed to the results of this study, no relationship was discovered between CTA and memory, compensation, and affective strategies. Besides, congruent with the findings of Ku and Ho (2010), this study found “good critical thinkers” are more active in meta-cognitive activities. Furthermore, this study proved Mahmoodi and Dehghannezhad’s (2015) findings that there was a significant and positive correlation between CTA and language learning strategies.

Regarding the mediating role of critical thinking abilities in the relationship between intermediate EFL learners’ learning strategies and their writing performances’, based on the statistical analysis, it was affirmed that CTA did not play a mediating role in the relationship between intermediate EFL learners’ learning strategies and their writing performances. As opposed to the researcher’s expectations, and as opposed to the fact that CTA was correlated with both learning strategies and writing performance, this study did not prove that CTA plays a significant role in the relationship between the other two variables. In other words, and surprisingly speaking, CTA does not guarantee the learners’ improvement in their writing performance.

## Conclusion

In the present research, an effort was made to examine the importance of CTA to learners in general and their writing output in particular. As stated earlier, this analysis concluded that there was a significant association between CTA and the writing achievement of EFL intermediate learners. In addition, there was a significant association between learning methods and writing achievements. Furthermore, there was a significant association between the six subgroups of learning strategies and writing results. It was concluded that all the sub-sets of cognitive, metacognitive, memory-related, social, affective, and compensation strategies were highly correlated with writing performance, meaning the use of these strategies would lead to a better performance in the task of writing. In addition, as opposed to what the researcher had envisaged, it was proved that although CTA had correlations with both writing performance and learning strategies, it did not play a mediating role in the relationship between learning strategies and writing performance of intermediate EFL learners.

The present study’s findings have shown that more concentration should be placed on critical thinking abilities to enhance students’ academic writing performance. Based on the outcomes of the study, it could be concluded that students benefiting from a very high level of CTA did better in their performances than those lacking such a high degree. The findings of this study demonstrate that the students could be more prosperous in their performances if they learn to think critically and if they are aware of the strategies. This attitude can be helpful for all Iranian English students who wish to be competent in perfect performances, especially in their writing performances.

The construction of CTA and learning methods has given rise to looking at teaching, training, and evaluation differently. Taking into account students’ needs, desires, and abilities, CTA pedagogy provides resources for authentic learning. The findings of this study depict that the students could be more successful writers if they boost their CTA and their learning strategy use. This can help all Iranian English students who long to be proficient in perfect performances in their writing tasks. In addition, educators can forecast effective language behaviors by defining CTA learner profiles at various stages of growth. Teachers must also understand that different CTA-level learners vary in their learning. Teachers might benefit from the study’s findings to realize their students’ levels of CTA, and their use of learning strategies would help them develop a sense of competence while being prepared for a performance. CTA is a vital tool that would alleviate writing performance and operate as a practical way to improve the quality of language learning.

In the first place, the results of this study can help teachers know learners’ plans in learning writing and assist their students in being responsive to use learning plans in their learning steps and creating a good learning context for using learning plans. Thus, students can become self-sufficient and accept responsibility for their learning. Secondly, curriculum developers may take advantage of the findings to include learning strategies training into the curriculum. As a result, students can use strategies in their learning process more efficiently. The current study can also assist in solving the problems of EFL teachers and learners in enhancing the level of cognitive and meta-cognitive abilities. The results may apprise educators that assisting learners in increasing their level of analysis and monitoring in learning is vital in learning.

## Data Availability Statement

The raw data supporting the conclusions of this article will be made available by the authors, without undue reservation.

## Author Contributions

All authors listed have made a substantial, direct, and intellectual contribution to the work, and approved it for publication.

## Conflict of Interest

The authors declare that the research was conducted in the absence of any commercial or financial relationships that could be construed as a potential conflict of interest.

## Publisher’s Note

All claims expressed in this article are solely those of the authors and do not necessarily represent those of their affiliated organizations, or those of the publisher, the editors and the reviewers. Any product that may be evaluated in this article, or claim that may be made by its manufacturer, is not guaranteed or endorsed by the publisher.
